# What is the impact of large-scale implementation of stroke Early Supported Discharge? A mixed methods realist evaluation study protocol

**DOI:** 10.1186/s13012-019-0908-0

**Published:** 2019-06-13

**Authors:** Rebecca Fisher, Niki Chouliara, Adrian Byrne, Sarah Lewis, Peter Langhorne, Thompson Robinson, Justin Waring, Claudia Geue, Alex Hoffman, Lizz Paley, Anthony Rudd, Marion Walker

**Affiliations:** 1 University of Nottingham, Nottingham, UK; 2 University of Glasgow, Glasgow, UK; 3 University of Leicester, Leicester, UK; 4 King’s College London, London, UK; 5 Guy’s and St Thomas’ NHS Foundation Trust, London, UK

**Keywords:** Stroke, Community-based rehabilitation, Early Supported Discharge, Realist evaluation, Implementation, Mixed methods

## Abstract

**Background:**

Stroke Early Supported Discharge (ESD) is a service innovation that facilitates discharge from hospital and delivery of specialist rehabilitation in patients’ homes. There is currently widespread implementation of ESD services in many countries, driven by robust clinical trial evidence. In England, the type of ESD service patients receive on the ground is variable, and in some regions, ESD is still not offered at all. This protocol presents a study designed to investigate the mechanisms and outcomes of implementing ESD at scale in real-world conditions. This will help to establish which models of ESD are most effective and in what context.

**Methods:**

A realist evaluation approach composed of two interlinking work packages will be adopted to investigate how and why ESD works, for whom and in what circumstances. Work package 1 (WP1) will begin with a rapid evidence synthesis to formulate preliminary realist hypotheses. Quantitative analyses of historical prospective Sentinel Stroke National Audit Programme (SSNAP) data will be performed to evaluate service outcomes based on the degree to which evidence-based ESD has been implemented. Work package 2 (WP2) will involve the qualitative investigation of purposively selected case study sites featuring in WP1 and covering different regions in England. The perspectives of clinicians, managers, commissioners, and service users will be explored qualitatively. Cost implications of ESD models will be examined using a cost-consequence analysis. Cross-case comparisons and triangulation of the data sources from both work packages will be performed to test, revise, and refine initial programme theories and address research aims.

**Discussion:**

This study will investigate whether and how current large-scale implementation of ESD is achieving the outcomes suggested by the evidence base. The theory-driven evaluation approach will highlight key mechanisms and contextual conditions necessary to optimise outcomes and allow us to draw transferable lessons to inform the effective implementation and sustainability of ESD in clinical practice. In addition, the methodological framework will progress the theoretical understanding of implementation and evaluation of complex rehabilitation interventions in stroke care.

**Trial registration:**

ISRCTN: 15568163, registration date: 26 October 2018.

Contributions to the literature

- Use of a realist evaluation framework with triangulation of quantitative and qualitative elements to investigate implementation of a complex intervention in a real world setting
- A methodological approach to investigate the dynamic nature between an intervention’s core components, actors delivering the intervention, and context to determine if trial-related outcomes of a complex intervention are realised in practice
- Use of national audit data and multilevel modelling statistics to explore effectiveness of a community-based stroke intervention at a patient and service level
- Analysis and data interpretation plans designed to inform future service implementation, local service improvement, and national performance monitoring

## Background

Stroke is one of the main causes of adult disability, and there is strong research evidence to show that provision of stroke specialist rehabilitation enhances recovery [1, 2]. Service delivery models that offer home-based stroke rehabilitation have gained increasing interest particularly as healthcare services face the challenge of cost reduction and integrated care provision [3–5].

Stroke Early Supported Discharge (ESD) is a multidisciplinary team intervention that facilitates discharge from hospital and delivery of stroke specialist rehabilitation at home and at an intensity equivalent to that provided in an acute stroke unit. Cumulative evidence from clinical trials has shown that ESD can reduce the length of hospital stay and the risk of dependency of stroke survivors [6, 7]. As a result, there is currently widespread implementation of stroke ESD services in the UK and other countries.

In England, National Clinical Guidelines from the Royal College of Physicians and the National Institute for Health and Care Excellence (NICE) recommend the provision of ESD as part of an evidence-based stroke care pathway [2, 8]. Yet, despite these research and policy drivers, national audit reports showed that the type of ESD services stroke patients receive is variable, and in some regions, ESD is still not offered at all [9]. Alternative models of operation have been adopted, but it is not known how close they are to the evidence-based models with demonstrated effectiveness in clinical trial settings. It remains unclear whether health and cost benefits of the ESD intervention are achieved when services are implemented in practice. With outcome-based commissioning becoming a priority within the National Health Service (NHS) in the UK, it is important to investigate whether ESD services are still effective in real-life clinical settings. Distinguishing between effective and ineffective implementation is crucial to address inequities in service provision and plan service improvements [10]. It is also vital with regard to informing implementation of sustainable evidence-based service models.

Large-scale implementation ESD needs to be considered in the light of the complexity inherent in the delivery of this type of intervention. ESD is a multidisciplinary and multicomponent healthcare intervention that involves a critical mass of stakeholders working across different organisation settings along the stroke care pathway. It is acknowledged that successful community-based care requires not only a shift in budget investment from acute to community services but also dissolving traditional occupation and organisational boundaries [3, 11]. ESD services do not operate in the controlled environments of experimental settings but in a complex and multilevel system such as the NHS. They are, therefore, exposed to a range of contextual influences at different levels of the healthcare system which act synergistically or antagonistically to evidence-based implementation. Decoding the observed variability requires distinguishing between receptive and non-receptive contexts as well as understanding the interplay between these environments and the programme’s ‘active ingredients’ [12]. This process of enquiry permits a better understanding of why the programme works in certain settings and not in others, what mechanisms underlie the programme’s success, and what steps we need to make in order to achieve the desired outcomes. Findings can then inform future implementation, reconfiguration, and improvement of ESD services to facilitate provision of evidence-based stroke care.

This paper presents the protocol of a multimethod study investigating what, why, and how ESD services are implemented and operate in real-world settings. The study responds to the second translation gap between clinical trials and clinical practice with the view to facilitating further implementation of ESD nationally and internationally. We will draw upon realist evaluation principles to unpick the interplay between ESD services and the context within which they operate, determine whether and how they are effective and in what conditions [13]. The study objectives will be as follows:

1. To investigate the effectiveness of ESD services when implemented at scale, in practice.
2. To understand how the context within which they operate influences the implementation and effectiveness of ESD schemes.
3. To identify transferrable lessons to drive effective implementation of stroke ESD in clinical practice.

To achieve these objectives, we will seek to answer the following questions:

1. What adopted models of ESD exist and how do these relate to evidence-based recommendations?
2. Can realised benefits of implementing ESD be quantified by defined measures of effectiveness: reduction in length of hospital stay, responsiveness of the service, amount of rehabilitation delivered, and changes in patient dependency?
3. What site-, model-, and patient-level characteristics influence effectiveness of ESD services?
4. What are the cost consequences of adopted ESD models?
5. What contextual elements influence whether ESD is implemented in the first place and how do they shape the model of service adopted?
6. What are the perceived outcomes of implementing ESD from the perspective of service users, clinicians, managers, and commissioners and how are these achieved in practice?
7. What are the conditions that contribute to the successful implementation and sustainability of ESD in practice?

## Methodology

### Conceptual framework

To address our research questions, we will draw on a realist evaluation (RE) approach [13]. RE is a theory-driven research evaluation that attempts to unpack the black box between complex healthcare programmes and the generated outcomes. Healthcare programmes are perceived as the manifestation of explicit or implicit theories that embody the developers’ and implementers’ assumptions about how the programme works [13, 14]. The evaluation starts by eliciting these key theories and mapping them into context-mechanisms-outcomes (CMO) configurations. CMOs are essentially hypotheses that explain what works, for whom, under what circumstances and how. In the process of data collection, realist hypotheses are put to test, revised, and refined, leading to more sophisticated programme theories and a better understanding of how programmes achieve their outcomes and in what settings [15].

We will start our inquiry by surfacing the core ‘formal programme theory’ about how ESD schemes work, as advocated by the evidence base [13–16]. Our previous research and national clinical guidelines have shed light on the ‘active ingredients’ that make an ESD service effective by defining evidence-based core components; these are the proposed essential characteristics that theory suggests need to be implemented for the programme to work in clinical practice [17, 18]. Formulated as a CMO proposition, what this evidence suggests is that ‘In urban settings (context), coordinated, stroke specialist multidisciplinary ESD teams (mechanisms - resources) provide timely hospital discharge and intensive home rehabilitation (outcomes), reducing length of in-hospital stay and improving long-term functional outcomes (outcomes)’.

Articulated in RE terms, it becomes apparent that the formal theory only partially explains how and in what contexts the programme works. In addition to patient level factors (i.e. stroke severity), previous research suggests that contextual elements operating at the levels of the team and the organisation as well as features of location need to be considered as part of an investigation of ESD services [17–19]. This fits well with current implementation research frameworks which highlight the importance of considering the characteristics of context at meso- and macro-levels [12, 20]. Since ESD is delivered to patients in their own home, thus necessitating the delivery of rehabilitation over potentially large geographical areas, the influence of the geographical location within which the service operates also needs to be understood. The question of how ESD schemes might operate in rural settings has been raised, given the fact that most of the original trials were conducted in urban settings. What needs to be stressed, however, is that the mere description of context does not explain why a different context generates different outcomes [21]. Examining the interaction between contexts and programme mechanisms will also be required in order to understand how the conditions within which the programme works activate and shape these mechanisms.

Regarding the underlying mechanisms, formal theory implies a causal relationship between the core components of the intervention and its outcomes [12]. According to the realist understanding of causation, interventions cannot directly cause outcomes but they provide (or take away) resources [15]. Programme mechanisms are understood as an interaction between the opportunities offered by the interventions and stakeholders’ reasoning and responses to these resources. Realists’ definition of mechanisms highlights the importance of human reasoning and interpretation as vital to understanding how an intervention works [14]. Evidence suggests that the behaviour of individual ESD team members, particularly across organisational boundaries, might influence the adoption and delivery of ESD services [19]. This also resonates with current implementation theory, which acknowledges the importance of actors involved in implementation as well as the context in which they are operating [20].

To fulfil the study’s aim, the formal theory needs to be refined and preliminary realist hypotheses developed, codified into CMO conjectures, and tested through data collection. We drew on the work of Dalkin et al. [22] who suggested that explicitly disaggregating mechanisms into resources and responses highlights the difference between the intervention and generative mechanisms and facilitates the formulation of CMO configurations. We conceptualised the evidence-based core components of ESD services as the programme resources, and we will seek to surface the perspectives and behaviours of actors and stakeholders (staff and patients) who are making ESD happen on the ground. As intended outcomes, we used the process and patient outcomes examined by clinical trials and the national stroke audit, but we also allowed for exploration of unintended outcomes, mainly through the qualitative component of the study. Table 1 presents an initial CMO framework, which we will use to configure our realist hypotheses and examples of initial plausible scenarios. This example explores how the rurality of the location of the ESD service may lead to a facilitatory or countervailing interaction with mechanisms to generate intended or unintended outcomes. These hypotheses will be put to test through data collection and analysis in order to fine-tune underpinning CMO configurations and increase our understanding of how these elements are related [13]. This approach will be compared to existing implementation research frameworks, acknowledging the dynamic nature between an intervention’s core components, actors delivering the intervention, and the context in which this takes place [12, 20].

**Table 1:** Programme theory CMO framework

Context	Mechanisms (resources + responses)		Outcomes
Rurality of ESD service location	Eligibility criteria		Accelerated transfer of care from hospital to home
	Team composition		
Commissioning and financial arrangements	Whole time equivalent staff/patient ratio	Staff perspectives/behaviour	Rehabilitation delivery—responsiveness
ESD provider organisation	Stroke specialism and staff training	Patient perspectives/behaviour	Rehabilitation delivery—intensity of rehabilitation
Referring services characteristics and locations	Multidisciplinary team co-ordination (e.g. meetings)		Patient outcomes—recovery

Example of a facilitatory CMO with intended consequences

‘If members of an ESD service have to cover long travelling distances in a rural setting, then the team may respond by making effective use of communication at team meetings and increased coordination (timetabling), resulting in patient centred goal setting and increased intensity of rehabilitation provided’

Example of a countervailing CMO with unintended consequences

‘If members of an ESD service have to cover long travelling distances in a rural setting, this may place a burden of the team’s communication and coordination and result in patients receiving less therapy than patients in urban settings’

### Study design

**Fig. 1:**
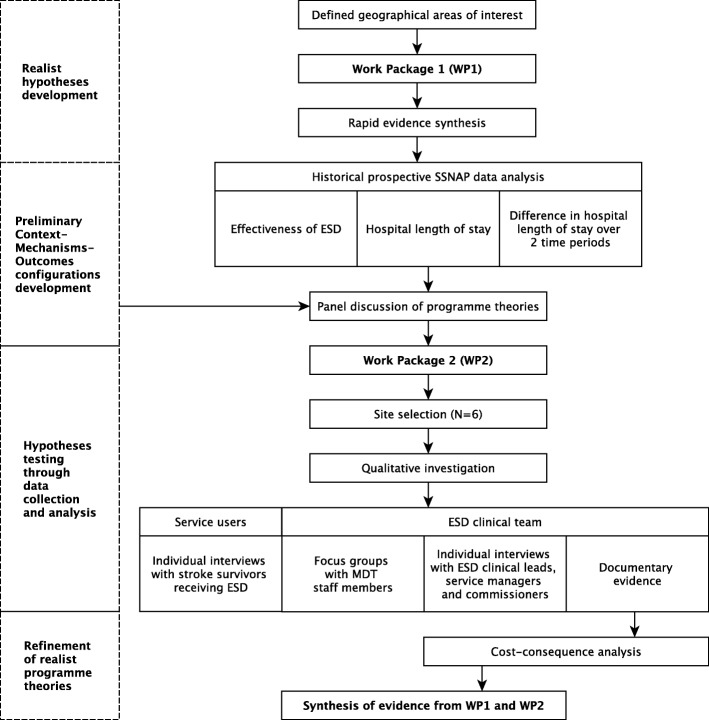
Flowchart of study including work packages 1 and 2

We will adopt a mixed methods design to draw information from multiple complementary sources. Our previous research and quantitative data analysis will inform mainly the ‘Context’, ‘Mechanisms-resources’ and ‘Intended Outcomes’ and qualitative data used to elicit ‘Mechanisms-responses’ and ‘Unintended outcomes’ [17–19]. The study will be conducted in two stages corresponding to two interlinking work packages undertaken sequentially (Fig. 1). Work package 1 (WP1) will begin with a literature review aiming to identify key contextual determinants to the implementation of ESD service and elicit potential mechanisms. Quantitative analyses of historical prospective Sentinel Stroke National Audit Programme (SSNAP) data will be then performed. The results from WP1 will complement but also feed into work package 2 (WP2) by informing the development of candidate realist programme theories formulated as CMO configurations which will then be tested, revised, and refined through WP2. Work package 2 will generate insights from the perspectives of actors and stakeholders who are making ESD happen in the real world. An exploratory multiple case study design will allow ESD sites featured in WP1 to be investigated qualitatively, drawing information from individual interviews, focus groups, and documentary evidence. A cost-consequence analysis will be performed to investigate the costs associated with ESD implementation drawing information from interviews and documentary evidence. Finally, data from each work package will be synthesised in order develop more sophisticated programme theories (with underpinning CMOs) that address the study’s questions.

### Work package 1

#### Rapid evidence synthesis

Given that context is a ‘slippery’ notion, identifying the salient contextual conditions relevant to the operation of ESD services will be the first step towards developing realist hypotheses. A rapid evidence synthesis (RES) will be conducted to identify contextual features having the potential to facilitate or impede the implementation of services providing home-based stroke rehabilitation [23]. RES is gaining popularity as it provides a robust and pragmatic approach to conducting a literature review to address specific questions. Database searches will be carried out in the Medline (OVID), CINAHL, and Embase databases for articles published since January 2000. A mixed research synthesis approach will be used where both qualitative and quantitative studies will be considered for inclusion based on their relevance to our research question. To define and structure different types of contextual factors identified in the literature, we will draw on implementation research models of determinants [12, 20, 24]. For the purposes of this study, we are particularly interested in the influence of adoption of evidence core components and the rurality of ESD service location.

#### Site selection

This study is designed to investigate the impact of different models of ESD operating over defined geographical regions of the East Midlands, West Midlands, East of England, and North of England (clinical network boundaries). This will allow an investigation of the consequences of a Midlands and East initiative to implement ESD beginning in 2012 [25] contrasted with a region (North of England) that has been slower to implement ESD based on SSNAP post-acute organisational audit data [9]. Sites within each of the four regions will be defined according to Clinical Commissioning Group (CCG) and local authority boundaries. All individual ESD teams within each site that participate in the SSNAP clinical audit will be included.

#### Data collection and analysis

Work package 1 will involve analysis of historical prospectively collected SSNAP data from hospital and community providers across the East Midlands, West Midlands, East of England, and North of England strategic clinical networks. A key aim of WP1 is to investigate whether the degree to which an ESD service has adopted an evidence-based model is related to better patient care (measured by ESD responsiveness and rehabilitation delivered) and patient dependency (modified Rankin Scale) [26] as well as the influence of rurality of the ESD service location. Effects of ESD on length of hospital stay will also be investigated; however, this analysis will require a different calibration of the statistical model and will be dealt with separately. To conduct these analyses, we employ multilevel modelling as it is an appropriate technique when analysing outcome variables that are generated from a clustered/nested structure whereby the outcome under consideration is produced by patients in different ESD team/hospital settings.

The effectiveness of ESD will be measured with evidence-based metrics as defined by national clinical guidelines for stroke and that reflect outcome measures used in the original ESD trials [6, 7]. Measuring the effects that ESD teams have on their patients is a necessary first step to learning how ESD practices combine to generate differences between teams. Combining SSNAP clinical audit data at the patient level with post-acute organisational audit data at the ESD team level, we will attempt to measure the ‘true’ effects that ESD teams have on their patients by fitting two-level patients-within-teams multilevel models to patient and process outcomes where covariate adjustments are made for a range of patient and ESD team characteristics.

#### Evaluating the effectiveness of ESD service provision

To investigate our study hypotheses, information about each ESD service collected by SSNAP in the 2015 post-acute organisational audit will be collated by the research team. This will include ESD team composition, capacity, workload, and organisational features, e.g. frequency of team meetings. ESD team models will then be analysed by comparing ESD service information with ESD consensus statements and national clinical guideline recommendations [2, 8, 17]. This will be the first step in the investigation of adoption of evidence-based core components, e.g. stroke specificity and multidisciplinary team composition. An ESD consensus score will be applied to each ESD team included in our multilevel modelling.

Additional variables in the model obtained from the 2016 SSNAP clinical data at the patient level will include age at admission, sex, pre-stroke independence, comorbidities, NIH Stroke Scale score on admission, type of stroke, and modified Rankin score at discharge from hospital [26, 27]. These reflect previously validated stroke case-mix models [28, 29]. Data variables at the ESD team level (in addition to the ESD consensus score) will include level of rurality and deprivation [30] as well as the grade/score of each discharging hospital as recorded by the SSNAP team (to account for the influence of the quality of inpatient care prior to ESD). A ‘team-size’ score will also be included (total whole time equivalent (WTE) units per member of staff). By including these ESD team-level ‘contextual variables’ in our analysis, we are assuming that they may not be homogeneous across the ESD teams and may impact upon service provision as well as patient outcomes. Moreover, multilevel modelling enables us to appreciate the variation in outcomes as a mixture of patient variability nested within ESD service provision variability. Effectiveness of ESD service provision will be measured with the following outcome variables: responsiveness (time from hospital discharge to first contact), rehabilitation intensity (total number of treatment days/total days with ESD), and stroke survivor outcome (modified Rankin Scale after ESD delivered).

#### ESD impact on patient length of hospital stay

A key benefit of ESD identified in the original randomised controlled trials was a reduction in length of hospital stay [6, 7]. Hence, we have also included this measure in our proposed analysis. Using our multilevel modelling framework and controlling for our covariates, this analysis will examine how patient length of hospital stay is influenced by the presence or absence of an ESD service on their care pathway.

Independent of the ESD team data, 2015–2016 hospital SSNAP data will be analysed to determine if patients from admitting hospitals that have ESD available as part of their care pathway differ in total length of inpatient stay from patients from admitting hospitals that do not, whilst controlling for confounding variables (e.g. patient level characteristics highlighted above). For this analysis, patients will be nested within admitting hospitals rather than ESD teams, and hence, different datasets will be used. Hospital characteristics of interest are type of hospital (e.g. as recorded by the SSNAP team) and a measure of delayed transfers of care from hospital, derived from the Adult Social Care Outcomes Framework (ASCOF), to account for influence of provision of social care [30]. The outcome variable for these analyses will be hospital length of stay at the patient level.

An additional analysis using a difference-in-difference, i.e. before-and-after design, will then be conducted to further investigate the magnitude of ESD impact on patient length of hospital stay [31]. This will provide a comparative analysis to the previous cross-sectional approach whereby we compare that 2015–2016 time period with a previous time period (2013–2014) in terms of ESD prevalence and its impact on inpatient length of stay across the two time periods. Difference-in-difference analysis enables investigation of differences in length of hospital stay over time and comparison between sites that have implemented ESD with sites that have not. To do this, the analysis requires use of data collected before and after the implementation of ESD. In this study, implementation of ESD is measured in terms of ESD being offered as part of the patients’ care pathway, from their admitting hospital and across the two time periods. Furthermore, we will determine which admitting hospitals had ESD as part of their care pathway at each time period, using patient-level SSNAP data, which tracks patient journey from first inpatient admission to final discharge destination.

### Work package 2

#### Site selection

Using a purposive sampling approach, case study sites from work package 1 will be selected based on the level to which evidence-based ESD has been implemented (contrasting ESD models) and the influence of rurality on ESD effectiveness (urban vs. rural sites).

#### Recruitment and interviews

##### Staff interviews

Semi-structured one-to-one interviews will be conducted with up to eight NHS staff informants at senior management, service lead, and commissioning level at each ESD site. Stakeholders (e.g. commissioners, ESD team leads) will be identified through collaboration with the national audit team and stroke clinical leads operating in each region. We will also liaise with NHS senior staff, invited to be part of our steering group panel, to ensure we identify and engage stakeholders effectively. Semi-structured interviews will allow us to explore individual stakeholder perceptions on a one-to-one basis, using a topic guide and prompts informed by our programme theories. These ‘realist’ interviews will be designed to expose individual stakeholder perspectives on the mechanisms involved in the implementation, delivery, and effectiveness of ESD and how these relate to contextual factors and desired outcomes.

##### ESD team interviews

We will also conduct two group interview sessions at each site. Two sessions will ensure a representative sample of the ESD team (physician, nurses, therapists, rehabilitation assistants, etc.) is included each time without disrupting provision of patient care. The aim will be to facilitate group discussion to uncover shared group perspectives on how and why the ESD service operates as a whole and how this relates to performance and sustainability. Teams will be given the opportunity to reflect on contextual conditions and processes that they perceive contribute to service effectiveness and to consider how this relates to their environment and team make up. These will then be discussed in relation to their perspectives on effectiveness and the outcomes the service achieves. Should divergent views or conflict become apparent in the group, we will follow these up with one-to-one interviews with individual members of the team.

##### Stroke survivor interviews

Interviews with purposively selected ESD patients from each ESD site will also be conducted. Patients will be recruited by the ESD teams in consultation with the research team, selecting patients currently on their caseload. Purposive sampling will allow the means to ensure the sample includes patients with a variety of experiences. Patient interviews will focus on areas such as experience of rehabilitation at home, staff interaction, and what aspects of ESD mattered most to them. Interviews will explore what patients believe the purpose of ESD to be, how ESD services should be monitored, and how services could be improved and why. These data will provide an important perspective with regard to active participation in mechanisms relating to the delivery of ESD and outcomes achieved.

#### Documentary evidence

To investigate contextual factors that stakeholders may not readily articulate and understand the operation costs of each service, we will also gather documentary evidence. This will be in the form of service specifications, monthly and annual reports, meeting notes, and paperwork used by the teams as part of their day-to-day operational activities. Costs will be explored using forms developed for the purpose to be completed by service managers.

#### Qualitative analysis

Data will be analysed iteratively, following a retroductive approach [15]. Participants’ narratives and documentary data from each site will be examined to identify connections between contexts mechanisms and outcomes, coded into CMO strings [32]. An overarching framework will be developed which is informed by ‘a priori’ issues including important differences in the case study sites selected (rural/urban, ESD model categorisation) and will be used to group CMOs in three levels: staff member/service user level, ESD team, and location or site. Cross-case comparisons will then be conducted to identify confirming and disconfirming cases and explore how pertinent mechanisms interact with site-specific contextual conditions to generate variation in outcomes. The identified CMO configurations will be related back to the original programme theories, and further refinements will be made.

#### Cost-consequence analysis

Cost implications are likely to be an important consideration with regard to successful adoption and implementation of interventions such as ESD. Economic evaluation methods for complex interventions, such as ESD, should ideally consider the wider costs and benefits associated with the intervention. It has been argued that generic outcomes such as quality-adjusted life years (QALYs) may not be suitable to capture these wider effects [33]. In addition, standard cost-effectiveness measures have poor recognition of the importance of context, lacking interest in links between causal mechanisms and relevant contexts that are thought to produce expected outcomes. Resources associated with delivery of a complex intervention are likely to equate to different costs in different places [34].

Given these issues, a cost-consequence analysis will be deployed. This form of analysis has been recommended for complex interventions that have multiple effects as it offers a more flexible approach to presenting costs and benefits alongside each other rather than combing these into a single measure [33]. This approach also fits with the realist approach of the study, in that costs associated with ESD-related mechanisms will be considered in light of context in which they are operating and the outcomes or benefits that are achieved.

In order to obtain the cost data necessary for an analysis of this kind, contrasting ESD model types represented by the six teams selected for inclusion in work package 2 will be used. SSNAP post-acute organisational audit data will be supplemented by more detailed information gathered from teams directly (e.g. service specifications). Multidisciplinary team composition and workload (WTE) information will be used to calculate associated NHS costs. Staff training budgets will be considered (given the importance of stroke specific expertise). Travel costs associated with delivery of rehabilitation will be estimated by defining the geographical area over which the ESD service operates, determining average distances travelled and number of patient visits made. Using patient caseload information, direct costs per patient will also be calculated. Differences in length of hospital stay and changes in modified Rankin derived from the comparison of ESD model types will be used to estimate treatment effects.

#### Triangulation of findings from work packages 1 and 2

Triangulation of findings from work packages 1 and 2 will be conducted when both datasets have been analysed separately. CMO patterns from each work package will be compared and modification of programme theories reviewed. A convergence coding matrix will then be used to display findings emerging from each work package [35]. The triangulation approach will allow identification of meta-themes across both work packages and permit research questions to be addressed from different perspectives (silence and dissonance between findings). This will allow exploration of context, mechanisms, and outcome configurations relating to the implementation and effectiveness of ESD as measured by the national audit and in relation to outcomes perceived to be important by stakeholders on the ground. Findings will be related to existing implementation frameworks (e.g. constructs within the i-PARIHS framework) to inform future research and also facilitation approaches focused on adoption, reconfiguration, and improvement of ESD services [12, 20].

#### Achieving rigour

The study responds to the call for evaluation designs that consider the complexity of the large scale service innovations in healthcare [36]. To achieve rigour in developing and reporting the study, the realist evaluation quality standards will be applied [15, 16]. In line with the requirements of a theory-driven analysis, we opted for a multimethod strategy [14]. Triangulation of multiple sources of evidence will permit the testing and refinement of programme theories and strengthen the construct validity of the study [37]. In WP2, validity will be enhanced through careful selection of information-rich cases that maximise variability and a good understanding and description of each context. Rather than considering perspectivism as limitation, the importance of including multiple voices from a range of different stakeholders is highlighted in realist evaluation [38]. The qualitative component of the study will seek to include a large and diverse sample of purposively selected stakeholders to ensure varied and contrasting perspectives are captured [39]. Regarding the external validity of the study, though it is accepted that the context-specific nature of the findings in realist research limits their quantitative generalisability [40, 41], the theory-driven nature of the enquiry enhances the transferability of the refined programme theories to other settings with similar characteristics [42, 43].

## Discussion

### Relevance to current NHS policy

Delivery of stroke care in the patient’s home has gained increasing attention particularly as healthcare services face the challenge of reducing costs [5]. The National Health Service Long-Term Plan makes recommendations for increased investment in community healthcare services and calls for implementation and further development of higher intensity care models for stroke rehabilitation [4]. This study will inform commissioning by robustly evaluating the effectiveness of services in real-world conditions. This research also addresses questions raised by a national policy drive for integrated care [44]. The provision of healthcare in the community requires integration of services across hospital and community boundaries. ESD is a multidisciplinary intervention that spans the hospital/community divide and underpins an integrated stroke care pathway. Under debate is the need for disease-specific services particularly in post-acute care and the merits of focusing on specific patient populations (e.g. mild to moderate stroke survivors) [19, 45]. In many areas of the UK, alternative models of ESD are being considered or have been adopted. It is important that the consequences of this variation are investigated. Outcome-based commissioning is also becoming a priority within the NHS in England, emphasising the importance of being able to measure the impact of healthcare services delivered.

### Impact

Designed to robustly measure effectiveness and identify key mechanisms that drive successful ESD service delivery, the study will offer transferable findings to influence provision of stroke care across the UK. Findings will influence the commissioning of stroke services and will help address inequity in service provision. The research could also influence provision of care in other countries, so that ultimately a much larger population of stroke survivors benefits from home-based rehabilitation. An intervention that facilitates early discharge from hospital and provides healthcare in the community is an innovation from stroke care that could be applied to other disease areas.

The study focuses on provision of care at a particularly distressing time, when stroke survivors leave hospital and face the consequences of stroke back at home. By investigating if and how trial-based patient benefits can be realised in practice, this study will address the gap between clinical trials and healthcare provision in real-world settings. Findings from this research will add to theories about how complex interventions can be successfully implemented in practice and inform debate around appropriate methodology for evaluating healthcare services in practice [46, 47].

## Data Availability

Not applicable

## Abbreviations

ASCOF: Adult Social Care Outcomes Framework
CCG: Clinical Commissioning Group
CMO: Context-mechanisms-outcomes
ESD: Stroke Early Supported Discharge
NHS: National Health Service
NICE: National Institute for Health and Care Excellence
QALYs: Quality-adjusted life years
RE: Realist evaluation
RES: Rapid evidence synthesis
SSNAP: Sentinel Stroke National Audit Programme
WP1: Work package 1
WP2: Work package 2
WTE: Whole time equivalent
